# Interactions of Body Weight Loss with Lipophilic Toxin Storage: Commentary

**DOI:** 10.1016/j.tjnut.2024.01.018

**Published:** 2024-01-18

**Authors:** Ronald Jandacek, Min Liu, Patrick Tso

**Affiliations:** Department of Pathology and Laboratory Medicine, University of Cincinnati Medical Center Reading Campus, Cincinnati, OH, United States

**Keywords:** food intake, red blood cell, blood lipophilic toxins, lipoproteins, enterohepatic circulation

## Abstract

A high incidence of obesity and surplus body fat has been observed in wealthy countries for many decades. It is generally recognized that these excesses contribute to serious disease states, including type 2 diabetes and cardiovascular diseases. On the other hand, the adipose tissue stores relatively safely many environmental lipophilic toxins. However, rapid weight loss mobilizes these toxins to the blood to be exposed to vital organs, such as the brain, lungs, and others. With the introduction of potent diabetic drugs causing rapid weight reduction, the question of mobilization of lipophilic toxins to the blood should be considered. In this commentary, we raised this mobilization of adipose tissue toxins to the readers. Also, we discussed how these toxins may be eliminated from the body through the use of nondigestible fat, such as olestra or lipase inhibitors, such as Xenical.

It has been reported by numerous authors that the lipophilic toxins in the body, such as persistent organic pollutants, are stored in the adipose tissue [1]. It has been a subject of considerable interest to physiologists and clinicians. Jackson et al. [2] also reported the storage of lipophilic toxins in the adipose tissue. This phenomenon is not only true for animals but also for humans. In an article by Ploteau et al. [3], they reported the level of persistent organic pollutants in the circulation and adipose tissue in French women with endometriosis. More recently, Malarvannan et al. [4] investigated the dynamics of the persistent organic pollutants in adolescents during weight loss. The cardiometabolic side effects of weight loss were studied with different degrees of weight loss. The toxic effects of high and low doses of persistent organic pollutants are considered by this interesting paper and the authors [5] raised the issue of how cognitive functions may be affected by the changing concentration of the lipophilic toxins in the blood [6]. Lastly, Kim et al. [6] studied the pathologic effects of dioxins and polychlorinated biphenyls (PCBs) in human obese subjects before and after dramatic weight loss.

A high incidence of obesity and surplus body fat has been observed in wealthy countries for many decades. It is generally recognized that these excesses contributed to serious disease states, including type 2 diabetes and cardiovascular diseases. Common and seemingly reasonable approaches to reduce body fat have had little success, and obesity-related diseases continue to increase.

Recent discoveries related to body fat and its depletion raise interesting and important questions that should be considered as research continues in this area. First, a class of compounds that significantly reducing body fat has resulted from explorations of ways to treat type 2 diabetes. These compounds, agonists of glucagon like polypeptide - 1 (GLP-1), appear to be a significant advance in the treatment of obesity and excess body fat. There are many reports of significant body weight reduction when these drugs are administered to treat diabetes [7,8]. Therefore, it seems that for the first time in the quest for means to reduce body weight significantly, a viable method is available. We can, therefore, expect to observe many patients reducing body fat levels by using this therapeutic approach.

The introduction of an efficacious obesity reduction drug should, however, not be pursued without considering the undesirable effects accompanying the loss of body fat. One such effect is the mobilization of lipophilic compounds that are stored in the body’s adipose tissue, chlorinated compounds, such as dichlorodiphenyltrichloroethane (DDT), dichlorodiphenyldichloroethylene, and PCBs [4,5,[9], [10], [11]].

Weight loss in a patient with toxic concentrations of PCBs from industrial contact resulted in significant metabolic effects consistent with the mobilization of PCBs from relatively benign fat depots to more sensitive sites [12]. In experimental rats, tissue concentrations of a chlorinated lipophile, hexachlorobenzene, were found to increase with weight loss, with simultaneous increases of the hexachlorobenzene in the brain [7]. This redistribution of lipophiles can presumably occur with a reduction in adipose tissue stores. These mobilized toxic lipophilic molecules then bind to red blood cell membrane phospholipids and travel in the blood to different sites, e.g., brain, breast (in females), lung, etc.

The weight loss-induced increase in the bioavailability of stored chlorinated lipophilic compounds entering the enterohepatic circulation was countered by introducing the nonabsorbable fatty acid ester of sucrose previously available as the food additive, olestra [7,12]. The absorption of dietary PCBs, DDT, and hexachlorobenzene was repeatedly demonstrated to be markedly reduced when olestra was included in the diet. The partition of these lipophiles into the nonabsorbable organic olestra phase and its subsequent fecal excretion provides a satisfactory mechanism to explain the observations. Unfortunately, olestra is no longer produced by the Proctor and Gamble Company. Another approach to get rid of these toxic lipophilic compounds is by the consumption of lipase inhibitor. One such inhibitor is orlistat, and it binds tightly to the lipase molecule, thus preventing it from exerting its lipolytic action hydrolyzing the luminal triacylglycerol molecules to free fatty acids and partial glycerides. Because orlistat is bound to lipase molecules, it is not absorbed by the gastrointestinal tract. The undigested triacylglycerol is then excreted in the feces, carrying the lipophilic toxins mobilized from the adipose tissue through weight reduction. Because orlistat is not absorbed by the small intestine into the body, it has negligible side effects.

In addition to this interference with the absorption of dietary lipophiles, olestra was also found to increase the excretion rate of lipophiles that were stored in the body after previous ingestion. This effect was first seen in measurements of the rate of fecal excretion of previously ingested cholesterol in rats [13]. There was no effect, however, of olestra on bile acid excretion. These observations were consistent with cholesterol dissolution in the nonabsorbable oil phase and interference with enterohepatic circulation.

This reduction of stored lipophiles during olestra consumption was observed both during isocaloric diets with maintenance of body weight and fat, and during hypocaloric diets when lipophile mobilization occurs from the adipose tissue [14]. In summary, there is ample evidence that lipophilic toxic substances can *1*) be stored in adipose tissue, *2*) be mobilized from adipose tissue during diet-induced fat loss, and *3*) be actively eliminated from the body by interfering with enterohepatic circulation using nonabsorbable dietary lipids.

The mobilization of organochlorine compounds stored in adipose tissue during periods of caloric deficit raises an important question about another class of toxic compounds that are currently the focus of environmental studies. There is accumulating evidence that synthetic compounds based on the carbon-fluorine bond are in human tissues with long half-lives [15]. These “forever chemicals” are the result of the manufacture of specific compounds designed to be used in a variety of products that take advantage of the unique properties of the carbon-fluorine bond.

Observations of organochlorines are generally consistent with the following metabolic pathway: *1*) ingestion along with dietary lipids; *2*) deposition of the parent compound into adipose tissue and the liver; *3*) metabolism of the parent compound in the liver and excretion as components of bile and bile acid micelles; and *4*) mobilization from adipose tissue during caloric deficit.

The metabolism of organofluorine compounds is undoubtedly more complex than that of the organochlorine compounds in the environment. This complexity may have several roots. First, there appear to be numerous classes of organofluorine compounds in the environment and, therefore, in human and animal tissues [[16], [17], [18]]. This variety in structure and chemical behavior results in metabolic pathways that depend on the physical properties and lipophilicity of the organofluorine. The solubility of one omnipresent compound, perfluorooctanoic acid, is a function of the lipophilic C-F bond and the ionized substance's polarity. It, therefore, is minimally solubilized in adipose tissue, in contrast with organochlorine compounds.

The use of the bile acid binding agent, cholestyramine, was found by Genuis et al. [19] to increase the excretion of perfluoro compounds in humans and they concluded that interruption of enterohepatic circulation was the mechanism of action, similar to that seen with olestra’s effect on polychlorinated compounds. Olestra did not alter perfluorooctanoic acid's absorption and enterohepatic circulation, although observations were not made beyond 2 wk after ingestion of the perfluorooctanoic acid [20]. Effects after longer periods were not investigated. It would also be interesting to see if olestra would affect the excretion and storage of perfluorinated compounds other than the readily hydrolyzed acid form.

The well-documented presence in humans of long-lived “forever chemicals,” chlorinated and fluorinated hydrocarbons, sets the stage for long-term detrimental health effects. The presence of halogenated compounds in adipose tissue is well established for chlorinated compounds, but less well studied for fluorinated compounds. It is likely that the storage and metabolism of the fluorinated compounds vary among this multicomponent class of compounds. These effects on health can be exacerbated if persons with these stored chemicals suddenly lose a lot of weight, thus mobilizing the compounds to more sensitive tissues. Release from fat depots to more active carriers in serum (e.g., bound to red blood cells) results in potentially short-term and profound long-term adverse effects. There is yet much to be understood about perfluoro compounds, as evidenced by the association of these compounds with weight regain after diet-induced weight loss [21].

Although there is this plausible mechanism for the interaction of body weight loss with halogenated hydrocarbon toxicity, there needs to be a better understanding of interventions that can counter the toxicity accompanying sudden and significant weight loss. As noted above, the use of agents to interfere with enterohepatic circulation has been shown to be beneficial. At this time, when efficacious weight loss protocols appear to be available, it seems prudent to determine what effects weight loss-induced mobilization of lipophilic toxins have on human health and what regimens can effectively reduce the stored levels and biological activity of these substances. It is conceivable to determine the circulating concentration of the lipophilic toxins in patients undergoing weight loss regimens before and after the beginning of drug treatment.

## Author contributions

The authors’ responsibilities were as follows – RJ, ML, PT: contributed equally to the writing, editing, and finalizing of this commentary; had numerous discussions before the writing of the commentary and later regarding the content of this commentary; read the rebuttal to the reviewers' comments, the modified commentary as well as the acknowledgment; and approved the revision and their initials represent their approval of the revision and the revised paper.

### Conflict of interest

The authors report no conflicts of interest.

### Funding

The authors reported no funding received for this study.
